# Reliability of Smartphone for Diffusion-Weighted Imaging–Alberta Stroke Program Early Computed Tomography Scores in Acute Ischemic Stroke Patients: Diagnostic Test Accuracy Study

**DOI:** 10.2196/15893

**Published:** 2020-06-09

**Authors:** Kenichiro Sakai, Teppei Komatsu, Yasuyuki Iguchi, Hiroyuki Takao, Toshihiro Ishibashi, Yuichi Murayama

**Affiliations:** 1 Department of Neurology The Jikei University School of Medicine Tokyo Japan; 2 Department of Neurosurgery The Jikei University School of Medicine Tokyo Japan

**Keywords:** smartphone app, DWI, ASPECTS

## Abstract

**Background:**

High-quality neuroimages can be viewed using a medical app installed on a smartphone. Although interdevice agreement between smartphone and desktop PC monitor was found to be favorable for evaluating computed tomography images, there are no interdevice agreement data for diffusion-weighted imaging (DWI).

**Objective:**

The aim of our study was to compare DWI interpretation using the Join smartphone app with that using a desktop PC monitor, in terms of interdevice and interrater agreement and elapsed interpretation time.

**Methods:**

The ischemic change in the DWI of consecutive patients with acute stroke in the middle cerebral artery territory was graded by 2 vascular neurologists using the Join smartphone app and a desktop PC monitor. The vascular neurologists were blinded to all patient information. Each image was categorized as either Diffusion-Weighted Imaging–Alberta Stroke Program Early Computed Tomography Scores (DWI-ASPECTS) ≥7 or DWI-ASPECTS <7 according to the Japanese Society for Neuroendovascular Therapy. We analyzed interdevice agreement and interrater agreement with respect to DWI-ASPECTS. Elapsed interpretation time was compared between DWI-ASPECTS evaluated by the Join smartphone app and a desktop PC monitor.

**Results:**

We analyzed the images of 111 patients (66% male; median age=69 years; median National Institutes of Health Stroke Scale score on admission=4). Interdevice agreement regarding DWI-ASPECTS between the smartphone and the desktop PC monitor was favorable (vascular neurologist 1: κ=0.777, *P*<.001, vascular neurologist 2: κ=0.787, *P*<.001). Interrater agreement was also satisfactory for the smartphone (κ=0.710, *P*<.001) and the desktop PC monitor (κ=0.663, *P*<.001). Median elapsed interpretation time was similar between the smartphone and the desktop PC monitor (vascular neurologist 1: 1.7 min vs 1.6 min; *P*=.64); vascular neurologist 2: 2.4 min vs 2.0 min; *P*=.14).

**Conclusions:**

The use of a smartphone app enables vascular neurologists to estimate DWI-ASPECTS accurately and rapidly. The Join medical smartphone app shows great promise in the management of acute stroke.

## Introduction

In 1996, the first-line treatment for acute-onset ischemic stroke was intravenous thrombolysis using recombinant tissue plasminogen activator (IV rtPA) therapy, which was effective only within 3 hours after the onset of symptoms. Since then, the emergency medical systems for acute stroke patients have improved dramatically. The publication of the ECASS III (European Acute Stroke Study III) [1], DAWN (Clinical Mismatch in the Triage of Wake Up and Late Presenting Strokes Undergoing Neurointervention With Trevo) [2], and DEFUSE 3 (Diffusion and Perfusion Imaging Evaluation for Understanding Stroke Evolution) [3] trials and advances such as those reported from the WAKE-UP (Wake-Up Stroke) trial [4] and new thrombolytic agents [5] have expanded the therapeutic time window and increased the number of candidates suitable for IV rtPA and mechanical thrombectomy. Accordingly, the decision-making process for thrombolysis requires timelier, more accurate, and more professional neurological assessment (including neuroimaging) to be made by a stroke specialist. The sharing of clinical and neuroimaging information will become increasingly important in decision making for IV rtPA and mechanical thrombectomy, particularly at comprehensive stroke centers. There is an urgent need to build a more convenient and faster communication system for sharing this information among the stroke team, which comprises vascular neurologists, on-call physicians, residents, and emergency, operating room, and paramedical staff.

**Figure 1:**
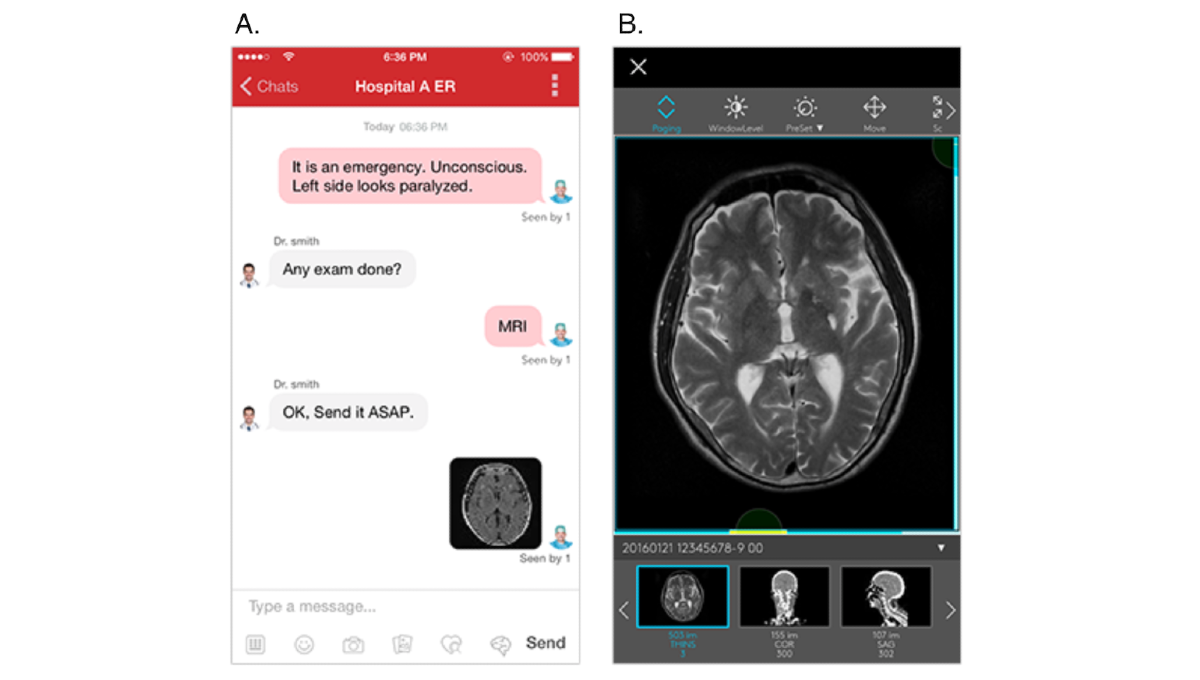
A. The Join smartphone app utilizes the easy-to-use interface of the social networking communication environment. B. Communication with picture archiving and communication system and other intrahospital systems enable text and medical images hosted on a cloud server to be shared in a group chat. The Join smartphone app displays diagnostic medical images, such as MRI and CT, and enables app users to edit, comment on, and draw a shape. CT: computed tomography. MRI: magnetic resonance imaging.

We used the Join medical smartphone app to build a seamless communication system for the stroke team. The app enables the team to share texts, neuroimaging, photos, and videos with high security (Figure 1A). Immediately after neuroimaging of a stroke patient, the images are sent from the hospital server to all smartphones that have the app installed and are signed in as members of the stroke team. The images can be enlarged and evaluations recorded with a simple touch sequence on the smartphone screen (Figure 1B). Before this communication system can be used in the newly extended therapeutic window in acute stroke, it is necessary to confirm interdevice agreement and interrater agreement with regard to assessment of the neuroimages.

At our stroke center, the initial imaging examination for patients with acute ischemic stroke is magnetic resonance imaging (MRI) rather than computed tomography (CT), for the following reasons: (1) hyperacute ischemic stroke is easily diagnosed on diffusion-weighted imaging (DWI) on MRI and (2) cerebral artery occlusion can be assessed on magnetic resonance angiography without the use of contrast agents. Although interdevice agreement between the smartphone and a desktop PC monitor was found to be favorable for evaluating CT images [6], there are no interdevice agreement data for DWI. The aim of our study was to compare DWI interpretation using the Join smartphone app with that using a desktop PC monitor, in terms of interdevice and interrater agreement and elapsed interpretation time.

## Methods

### Patient Characteristics

From January 2016 to September 2017, we enrolled 111 patients with acute ischemic stroke in the middle cerebral artery territory, diagnosed within 24 hours of onset. DWI on MRI was performed on all patients, and the following clinical information was recorded: cardiovascular risk factors (hypertension, diabetes mellitus, dyslipidemia, and smoking status) and atrial fibrillation. Stroke severity on admission was assessed using the National Institutes of Health Stroke Scale (NIHSS) score. Stroke subtype was categorized into four groups: small-vessel occlusion, large-artery atherosclerosis, cardioembolism, or other.

### Join Smartphone App

We evaluated the Join smartphone app (Allm Inc.), which was developed for use as a telemedicine app for health care professionals. The Join smartphone app leverages the easy-to-use interface of the social networking communication environment, such as SMS text messaging (Figure 1A) and, importantly, enables the stroke team to immediately share medical information such as diagnostic images (CT, MRI, and ultrasonography) and electrocardiograms as well as the results of blood tests (Figure 1B). Information sharing begins as soon as the emergency department is informed of an incoming potential stroke patient and continues as relevant personnel are called; the initial diagnostic and therapeutic orders are prepared, and senior staff are consulted if necessary. Following the acquisition of imaging studies, the images and radiological reports are shared. Additional details of the patient evaluation (including digital video recordings of clinical signs) can be requested and sent to the senior consulting staff. Following discussion among the team, the final management decisions are made before the patient is admitted to the stroke care unit. For purposes of security, no information is stored on any smartphone, and the app displays only the medical information and images that are streamed from the cloud server. On completion of the communication session, no discussion related to the patient remains on the smartphone.

### Imaging

DWI was performed with a 1.5 T MRI unit (MAGNETOM Avanto/MAGNETOM Symphony, Siemens) using the following sequence: repetition time/echo time=2700/90 ms, section thickness=5 mm, section gap=1.5 mm, matrix=128x128, field of view=21 cm. Diffusion-Weighted Imaging–Alberta Stroke Program Early Computed Tomography Scores (DWI-ASPECTS) were defined using a scoring template comprising 2 axial DWI slices with markers for 10 anatomical regions [7,8]. We checked the entire sequence of DWI slices to calculate the score. Each patient was categorized as DWI-ASPECTS ≥7 or DWI-ASPECTS <7, according to the statement of the Japanese Society for Neuroendovascular Therapy for patients undergoing mechanical thrombectomy.

**Figure 2:**
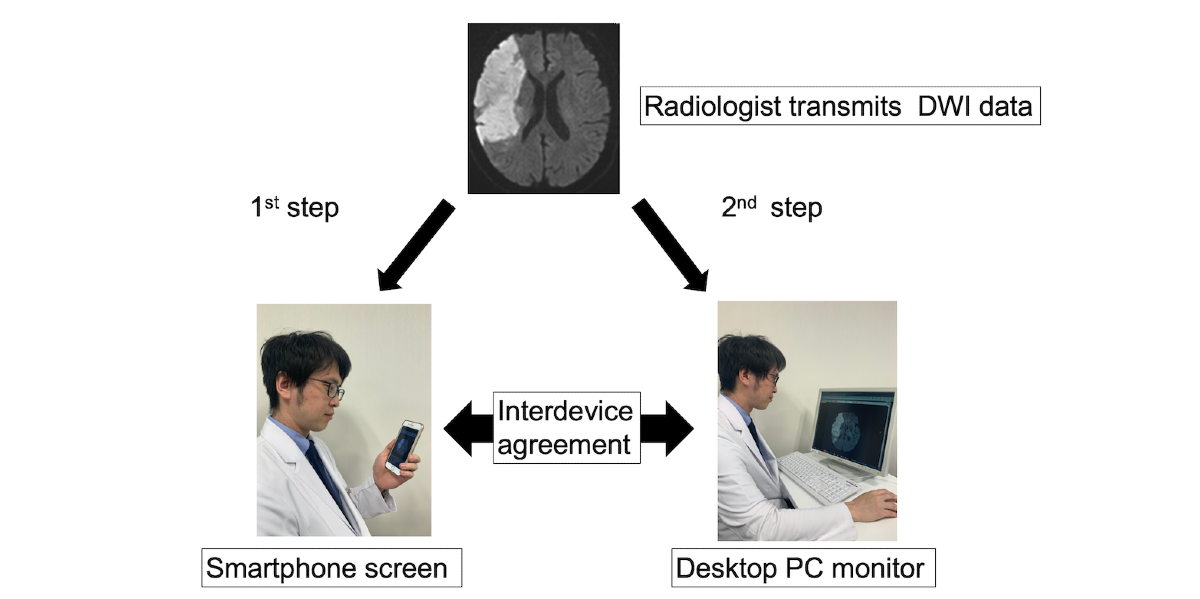
Study protocol. First, the vascular neurologists evaluated DWI-ASPECTS on a smartphone screen. Second, they evaluated DWI-ASPECTS on a desktop PC monitor. Interdevice agreement and interrater agreement were calculated for the same and for different devices. DWI-ASPECTS: Diffusion-Weighted Imaging–Alberta Stroke Program Early Computed Tomography Scores.

The study protocol was as follows (Figure 2). Two vascular neurologists (KS and TK) installed the Join smartphone app on their smartphones. After confirming operation of the app, a radiologist transmitted the DWI data of all patients to the vascular neurologists, in random order. As the first step, the vascular neurologists received the DWI data on their smartphones and independently scored DWI-ASPECTS for all patients using the Join smartphone app (JOIN-ASPECTS). The time for transfer of the DWI data was a few seconds. As the second step, vascular neurologists interpreted all DWI data on a desktop PC monitor and individually scored DWI-ASPECTS (PC-ASPECTS) a few days later. The vascular neurologists were blinded to the background and clinical information of all patients. We recorded the time taken for each vascular neurologist to complete DWI scoring for JOIN-ASPECTS and PC-ASPECTS**.** This study conformed to the ethical principles established in the Declaration of Helsinki, and the Institutional Review Board at the Jikei University School of Medicine approved the study protocol (no. 8813).

### Statistical Analysis

To calculate interdevice (smartphone and desktop PC monitor) agreement, we prepared scatter diagrams for JOIN-ASPECTS and PC-ASPECTS. Scatter diagrams were created for all patients, including patients with major arterial occlusion and those without major arterial occlusion. Kappa statistics were used to calculate interdevice and interrater (vascular neurologists KS and TK) agreement.

We defined a component of interrater agreement among the vascular neurologists as the agreement rate of DWI-ASPECTS in each patient, for a DWI cutoff of ≥7 or <7. We evaluated interrater agreement between the vascular neurologists for the smartphone, the desktop PC monitor, and then for both the smartphone and the desktop PC monitor. Interrater agreement was assessed using 2x2 cross-tabulation.

Kappa scores were rated as follows: <0.20, poor agreement; 0.21-0.40, fair agreement; 0.41-0.60, moderate agreement; 0.61-0.80, favorable agreement; and 0.81-1.0, almost perfect agreement. *P*<.05 was considered significant. All statistical analyses were performed using SPSS for Windows, version 22.0 (IBM Corp.).

## Results

**Figure 3:**
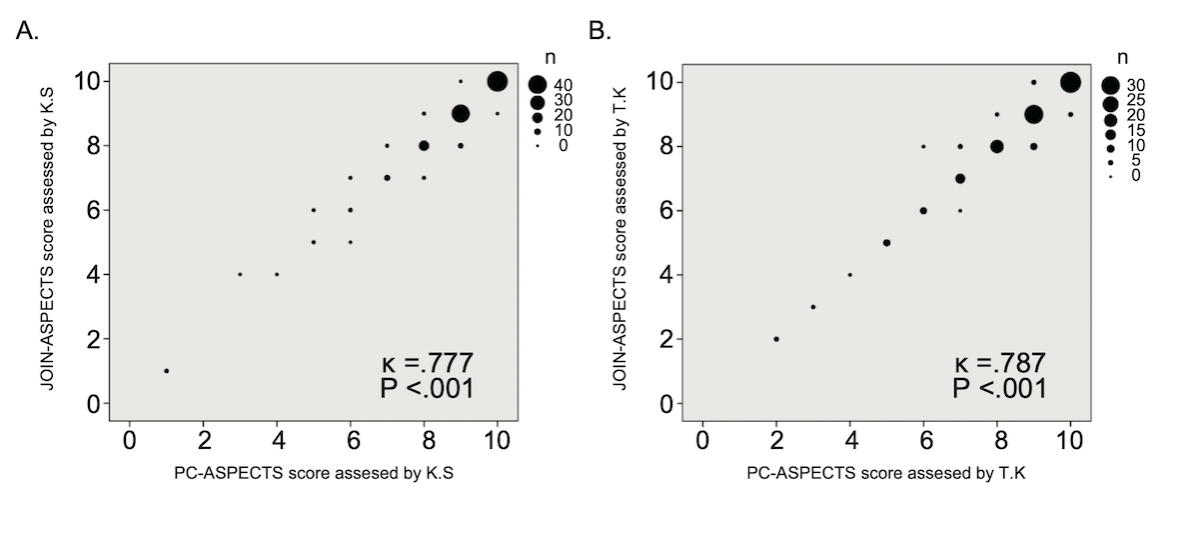
Scatter diagram of the DWI-ASPECTS results of vascular neurologists KS (A) and TK (B) between JOIN-ASPECTS and PC-ASPECTS for all patients. DWI-ASPECTS: Diffusion-Weighted Imaging–Alberta Stroke Program Early Computed Tomography Scores.

**Figure 4:**
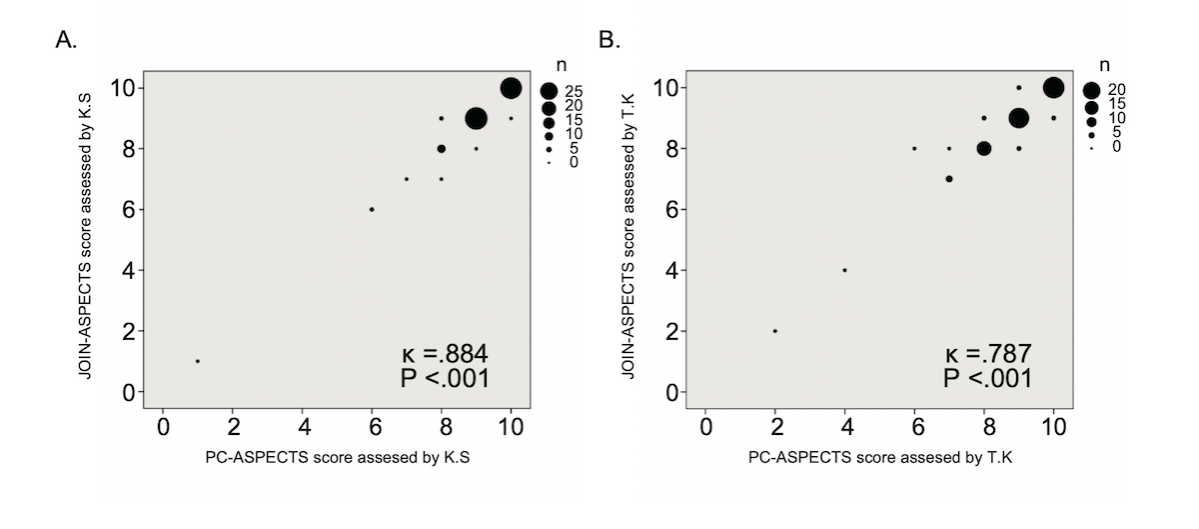
Scatter diagram of DWI-ASPECTS results of vascular neurologists KS (A) and TK (B) between JOIN-ASPECTS and PC-ASPECTS for patients without major artery occlusion. DWI-ASPECTS: Diffusion-Weighted Imaging–Alberta Stroke Program Early Computed Tomography Scores.

**Figure 5:**
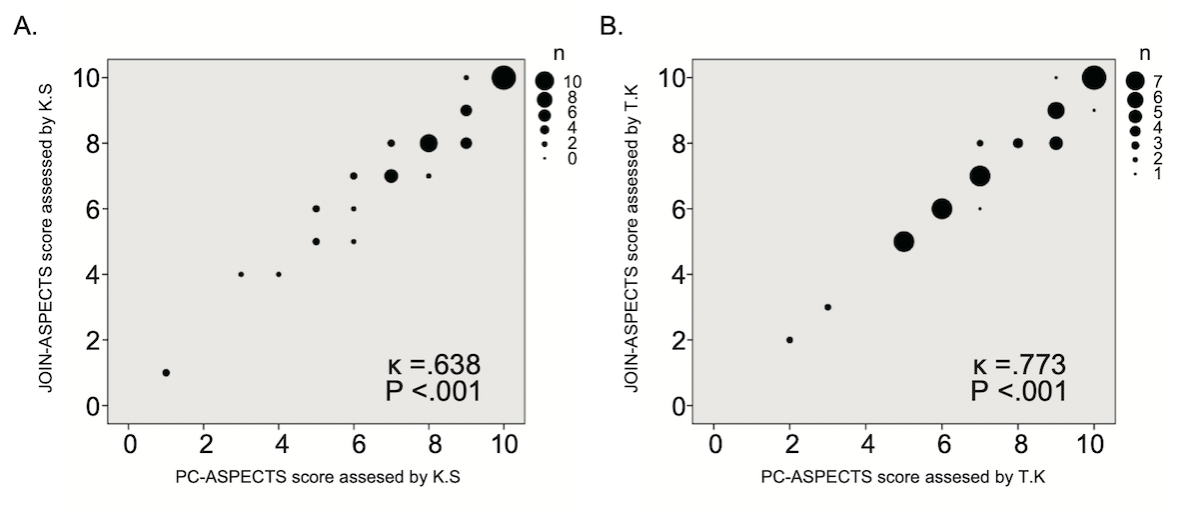
Scatter diagram of DWI-ASPECTS results of vascular neurologists KS (A) and TK (B) between JOIN-ASPECTS and PC-ASPECTS for 46 patients with major artery occlusion. DWI-ASPECTS: Diffusion-Weighted Imaging–Alberta Stroke Program Early Computed Tomography Scores.

We enrolled 111 patients (66% male; median age, 69 years; median NIHSS score on admission, 4). Table 1 lists the patients’ clinical characteristics. Median DWI-ASPECTS was 9 (6-10), and 46 (41%) patients had major artery occlusion. The median elapsed time between symptom onset and DWI imaging was 270 min. Interdevice agreement between the smartphone and the desktop computer monitor was favorable (KS: κ=0.777, *P*<.001; TK: κ=0.787, *P*<.001) for all patients (Figures 3A and 3B). Interdevice agreement was also favorable for patients with and those without major arterial occlusion (Figures 4A, 4B, 5A, and 5B). The median elapsed interpretation times (from receiving the image to finishing interpretation) were similar for the Join smartphone app and the desktop PC monitor (KS: 1.7 min vs 1.6 min, *P*=.64; TK: 2.4 min vs 2.0 min, *P*=.14). Interrater agreement between the 2 vascular neurologists was favorable for the Join smartphone app (κ=0.710, *P*<.001; Multimedia Appendix 1) and the desktop PC monitor (κ=0.663, *P*<.001; Multimedia Appendix 2). Interrater agreement was also favorable between the 2 vascular neurologists for the different devices (KS using the Join smartphone app and TK using the desktop PC monitor: κ=0.663, *P*<.001; KS using the desktop PC monitor and TK using the Join smartphone app: κ=0.723, *P*<.001; Multimedia Appendices 3 and 4).

**Table 1:** Patient characteristics (n=111).

Characteristic		Value
Age (years), median (IQR^a^)		69 (58-78)
Male, n (%)		73 (66)
**Past history, n (%)**		
	Hypertension	74 (68)
	Hyperlipidemia	49 (44)
	Diabetes mellitus	26 (23)
	Atrial fibrillation	22 (20)
NIHSS^b^ score on admission, median (IQR)		4 (2-7)
**TOAST^c^** **classification, n (%)**		
	Large-artery atherosclerosis	8 (7)
	Small-vessel occlusion	15 (14)
	Cardioembolism	35 (32)
	Other determined etiology	9 (8)
	Undetermined	43 (40)
mRS^d^ at 3 months, median (IQR)		1 (1-3)
**Imaging**		arge-artery atherosclerosis
	DWI-ASPECTS^e^, median (IQR)	9 (6-10)
	Major arterial occlusion, n (%)	46 (41)
	MRI^f^ time from onset, median (IQR)	270 (185-335)

^a^IQR: interquartile range.

^b^NIHSS: National Institutes of Health Stroke Scale.

^c^TOAST: Trial of ORG 10172 in Acute Stroke Treatment.

^d^mRS: Modified Rankin Scale.

^e^DWI-ASPECTS: Diffusion-Weighted Imaging–Alberta Stroke Program Early Computed Tomography Scores.

^f^MRI: magnetic resonance imaging.

## Discussion

### Principal Findings

There were 3 major findings in this study. First, there was a high degree of interdevice and interrater agreement in terms of the vascular neurologists’ neuroimaging findings between the smartphone and the desktop PC monitor among patients with acute stroke. Second, DWI-ASPECTS was favorable for smartphone–desktop PC monitor, desktop PC monitor–desktop PC monitor, and smartphone–smartphone. Third, the elapsed interpretation time for DWI-ASPECTS using the smartphone was similar to that for the desktop PC monitor. The smartphone was comparable to the desktop PC monitor concerning DWI in acute ischemic stroke patients.

Our study presents the following 2 original points. First, we evaluated neuroimaging using 2 different devices, a smartphone, and a desktop PC monitor. Telemedicine has been proposed for assessment and treatment of acute stroke patients, but few studies have investigated the reliability of telemedicine for neuroimaging assessment.

Previous reports of hub-and-spoke type telemedicine networks have shown the validity and reliability of a telestroke neuroimaging system (desktop PC monitor–desktop PC monitor) in differentiating between ischemic stroke and hemorrhage stroke [9-11]. To the best of our knowledge, this study was the first to confirm the diagnostic accuracy of neuroimaging accessed using a smartphone app. In addition, we investigated the agreement of DWI-ASPECTS for ischemic lesions. Measurement of ischemic core volume is essential for hyperacute stroke therapy [2]. According to the findings of the DAWN [2] and WAKE-UP [4] trials, DWI imaging should feature more prominently in assessment of the suitability of hyperacute ischemic stroke patients for treatments such as thrombolysis. Therefore, DWI-ASPECTS is a useful and important scale in hyperacute stroke care. The score and the specific DWI cutoff value should be determined and shared before thrombolysis and mechanical thrombectomy.

Our results were in line with those of previous studies that reported almost perfect interrater agreement [12-14]. According to the findings of the DAWN and WAKE-UP trials, the therapeutic time for thrombolysis can be extended if a mismatch in visibility of a lesion is found between DWI and fluid-attenuated inversion recovery. Thrombolysis with perfusion imaging using a contrast agent, as in the DEFUSE 3 trial [3], is limited to some special comprehensive stroke centers.

DWI-ASPECTS was carefully evaluated and shared among the physicians who participated in this study. We expect that in the near future, assessment by DWI-ASPECTS and sharing of this information using the smartphone app will become commonplace in the management of hyperacute stroke patients.

It is crucial in telemedicine to have a high degree of interdevice agreement for DWI-ASPECTS between the smartphone and PC monitor scores. Sharing information such as neuroimaging among vascular neurologists, emergency department staff, and paramedical staff enables the medical team to deliver IV rtPA and mechanical thrombectomy in the shortest possible time.

In this study, there was excellent physician acceptance and a high level of satisfaction with the smartphone app system. One difference in this study was that our proposed neuroimaging telemedicine service can be conducted smoothly using various devices rather than being limited to the conventional PC–PC system. The security of personal patient details in the Join smartphone app enables the stroke team to safely and rapidly share information that is important for acute stroke therapy. Thus, we consider that the use of a medical smartphone app would change the manner of communication among the members of the stroke team.

Many physicians currently use smartphones daily as a part of their clinical examinations [15]. The main requirements of a telestroke consultation are fast and accurate neurological assessment by a stroke specialist; a review of brain imaging; and formulation of the diagnosis and treatment plan, including assessment of eligibility for standard thrombolytic therapy and endovascular devices. Telemedicine carries a large burden regarding investment in equipment, which typically includes two or more PCs, a web camera, network system, and software. The Join smartphone app could be used in place of all of these components, as a standalone tool or as an adjunct to existing telemedicine technology. The widespread use of smartphones, coupled with widely available health care apps, could enable the affordable expansion of telestroke networks. In addition to the Join smartphone app, numerous other videoconferencing and teleradiology apps are available in the smartphone app marketplace, many of which could facilitate telestroke consultation in the manner described here.

Our results revealed no significant difference in the time required for neuroimaging interpretation between the smartphone and desktop PC monitor systems. The main requirements of a telestroke consultation are rapid and accurate neurological assessment; review of neuroimaging; and formulation of the diagnosis and treatment plan, including IV rtPA and mechanical thrombectomy. We estimated that using a smartphone could take time for a neuroimaging evaluation, because this is a possibility due to a time lag resulting from connection issues. However, we found no delay in the time required for a neuroimaging review between the wireless connection by smartphone app and the wired connection by desktop PC monitor.

Smartphones are in common use by many physicians and already contribute to decisions made in medical treatment. We believe that the advantages of using a smartphone app are its portability and faster time to access to target neuroimages. Usually, stroke neurologists are not necessarily in front of a desktop or laptop computer. If an evaluation were requested, a stroke neurologist would need a certain amount of time to reach the computer, boot up the computer, log in to a network with a tight security system, and finally start browsing the images using a picture archiving and communication system. Using a smartphone app can solve the problem of the amount of time it takes a neurologist to evaluate neuroimaging by skipping these processes. Supplementation of the app with neuroimaging software has the potential to transform the smartphone into a complete tool for acute stroke evaluation. The Join smartphone app can play an important role in teleconsultation even when the stroke team comprises members who are located outside the hospital and who cannot access a desktop PC monitor.

There are several limitations in this study. First, the 2 vascular neurologists who evaluated the neuroimages have extensive clinical experience. It would be necessary to evaluate the interpretations of those physicians who have less experience. Second, we restricted our evaluation to neuroimaging of an anterior circulation stroke. It may be more difficult for vascular neurologists to detect a small infarction in the brain stem [16,17]. If applicable, axial and sagittal DWI should be routinely examined. Finally, our relatively high DWI-ASPECTS values compared with those of previous investigations [12-14] suggest the influence of high interdevice and interrater agreement in our results.

### Conclusion

The Join smartphone app enabled stroke neurologists to estimate DWI-ASPECTS accurately and rapidly. We demonstrated the usefulness of the app in facilitating management of acute stroke using a system that offers sharing of images and discussion across different devices, in the manner of a social networking service.

## Appendix

Multimedia Appendix 1 Table S1. Inter-rater agreement for DWI-ASPECTS ≥7 or DWI-ASPECTS <7 evaluated using the smartphone monitor.

Multimedia Appendix 2 Table S2. Inter-rater agreement for DWI-ASPECTS ≥7 or DWI-ASPECTS <7 evaluated on a desktop PC monitor.

Multimedia Appendix 3 Table S3. Inter-rater agreement for DWI-ASPECTS ≥7 or DWI-ASPECTS <7 for desktop PC monitor in VN1 vs smartphone monitor in VN2.

Multimedia Appendix 4 Table S4. Inter-rater agreement for DWI-ASPECTS ≥7 or DWI-ASPECTS <7 for smartphone monitor in VN1 vs desktop PC monitor in VN2.

Multimedia Appendix 5 CONSORT-EHEALTH checklist (V 1.6.1).
