# Supplementary material for: Reliability of Smartphone for Diffusion-Weighted Imaging–Alberta Stroke Program Early Computed Tomography Scores in Acute Ischemic Stroke Patients: Diagnostic Test Accuracy Study
Source: J Med Internet Res. 2020 Jun 9;22(6):e15893. doi: 10.2196/15893 (PMC7312257; doi:10.2196/15893)
Supplement: Multimedia Appendix 2 [file jmir_v22i6e15893_app2.pdf]

Table S2. Inter-rater agreement for DWI-ASPECTS ≥7 or DWI-ASPECTS <7 evaluated on a desktop PC monitor

		K.S		
		PC-ASPECTS<7	PC-ASPECTS≥7	
T.K	PC-ASPECTS<7	12	3	15
	PC-ASPECTS≥7	7	89	96
Total		19	92	111

ASPECTS: Alberta Stroke Program Early CT Score
JOIN-ASPECTS: ASPECTS on diffusion weighted magnetic resonance imaging using JOIN smartphone app
PC-ASPECTS: ASPECTS on diffusion weighted magnetic resonance imaging using desktop PC monitor
